# Linking the Wrangellia flood basalts to the Galápagos hotspot

**DOI:** 10.1038/s41598-021-88098-7

**Published:** 2021-04-21

**Authors:** J. Gregory Shellnutt, Jaroslav Dostal, Tung-Yi Lee

**Affiliations:** 1 Department of Earth Sciences, National Taiwan Normal University, 88 Tingzhou Road Section 4, Taipei, 11677 Taiwan; 2 Department of Geology, Saint Mary’s University, 923 Robie Street, Halifax, NS B3H 3C3 Canada

**Keywords:** Planetary science, Solid Earth sciences

## Abstract

The Triassic volcanic rocks of Wrangellia erupted at an equatorial to tropical latitude that was within 3000 km of western North America. The mafic and ultramafic volcanic rocks are compositionally and isotopically similar to those of oceanic plateaux that were generated from a Pacific mantle plume-type source. The thermal conditions, estimated from the primitive rocks, indicate that it was a high temperature regime (T_P_ > 1550 °C) consistent with elevated temperatures expected for a mantle plume. The only active hotspot currently located near the equator of the eastern Pacific Ocean that was active during the Mesozoic and produced ultramafic volcanic rocks is the Galápagos hotspot. The calculated mantle potential temperatures, trace elemental ratios, and Sr–Nd–Pb isotopes of the Wrangellia volcanic rocks are within the range of those from the Caribbean Plateau and Galápagos Islands, and collectively have similar internal variability as the Hawaii-Emperor island chain. The paleogeographic constraints, thermal estimates, and geochemistry suggests that it is possible that the Galápagos hotspot generated the volcanic rocks of Wrangellia and the Caribbean plateau or, more broadly, that the eastern Pacific (Panthalassa) Ocean was a unique region where anomalously high thermal conditions either periodically or continually existed from ~ 230 Ma to the present day.

## Introduction

The western North America Cordillera is an assemblage of allochthonous fault-bounded terranes that were juxtaposed with Laurentia and previously accreted terranes during the Paleozoic and Mesozoic^1–3^. However, the origin of the terranes themselves, the location of accretion zones, and the orientation of subduction zones responsible for closing the intervening ocean basins are still unresolved^4–8^. The westernmost part of the assemblage of terranes of the North American Cordillera is the Insular Superterrane which is composed of the Wrangellia and Alexander terranes. These two terranes may be related but are considered to be exotic to North America^9^.

**Figure 1:**
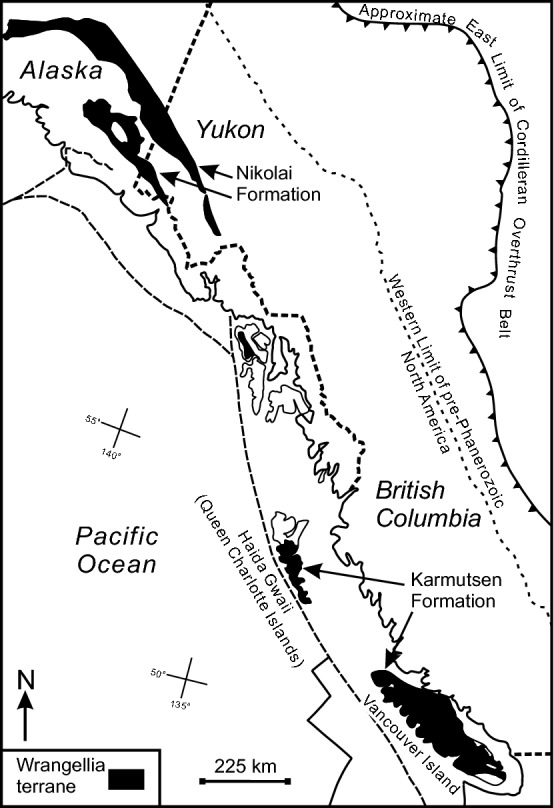
Location and distribution of the Wrangellia terrane^15^.

Wrangellia extends along the Pacific margin of North America from southern Vancouver Island through Haida Gwaii to southern-central Alaska (Fig. 1). The terrane is characterized by similar sequences of Triassic rocks. These rocks, dominated by thick, massive Middle to Upper Triassic tholeiitic flows and pillow lava (i.e., Nikolai Greenstone and Karmutsen Formation), overlie Devonian through Permian volcanic, volcaniclastic, siliciclastic, and carbonate rocks^1,10–13^. The volcanic rocks are thought to be derived from a “plume-type Pacific mantle source” that was similar to the Ongtong Java or Caribbean oceanic plateaux and may have contributed to the Carnian mass extinction^14–19^. In turn, Upper Triassic to Lower Jurassic reef carbonates and deep-water calcareous clastic strata and continentally derived sedimentary rocks of Lower Jurassic age^20^ conformably overlie them. At the well-exposed sections of Haida Gwaii, these units are overlain by the Middle-Upper Jurassic arc-volcanic and epiclastic rocks that are synchronous with extensive plutonism^21–24^.

There is a considerable uncertainty regarding the latitude of Wrangellia’s accretion to North America, its Late Cretaceous paleogeographic position, and the mechanism of accretion^5,8,25^. The debate, referred to as “Baja British Columbia”^26^, has fueled discussions for decades^27–30^. It is based on paleomagnetic data that suggests large parts of western North America, specifically Wrangellia, were located in present day southern California/Mexico (Baja) during the Late Cretaceous^27^. The paleomagnetic data^26,31–36^ requires that Wrangellia was transported 2000–3000 km northward to its present location over a period of ~ 20 Ma^27^. Detrital zircon studies also support large-scale translation during the Late Cretaceous and Early Paleogene^37,38^. However, there is no clear field evidence of geological structures necessary to accommodate large-scale displacements of the Insular Superterrane. This lead to some doubts of the validity of the paleomagnetic data^23,39,40^. Tectonic models based on mapped strike-slip faults east of the Insular Superterrane limit displacement to < 900 km northward whereas paleomagnetic estimates vary from ~ 1000 to > 3000 km^28,34,41,42^. In essence, the dispute is that the northward translation called for by the paleomagnetic results is larger than has been accounted for by geologic studies.

In this paper we evaluate the possible correlation of the Triassic flood basalts of Wrangellia to an East Pacific equatorial hotspot. Specifically, we attempt to link the generation of the Wrangellia volcanic rocks to the Galápagos hotspot based on the paleomagnetic data, and whole rock and radiogenic isotopic compositions (Sr–Nd–Pb). The geochemical data are used to calculate mantle potential temperatures of the primitive Wrangellia flood basalts, and the Sr–Nd–Pb isotopes are used as a comparison to the mantle source characteristics of rocks generated from the Galápagos hotspot since the Cretaceous.

### Paleogeography, composition and thermal history of the Wrangellia flood basalt

**Figure 2:**
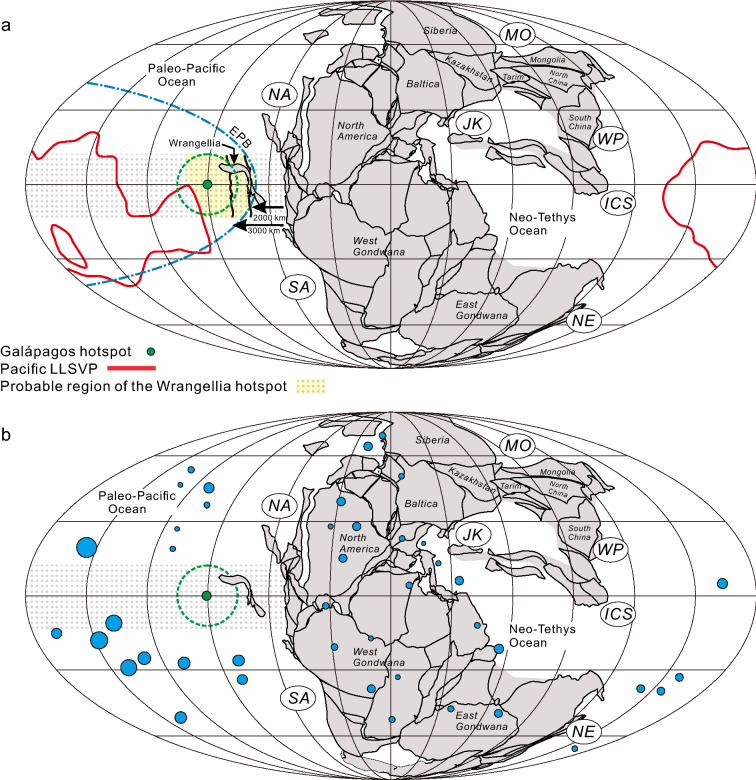
(**a**) Late Triassic paleogeographic reconstruction showing the location of Wrangellia with respect to the permitted paleomagnetic latitudinal range, the Pacific LLVSP^63^, the Eastern Pacific Ocean boundary (EPB)^58^, and the current location of the Galápagos hotspot^52^. The green dashed circle is the permitted plate motion radius of the Galápagos hotspot (± 15°). (**b**) Late Triassic paleogeographic reconstruction showing the locations of the Galápagos hotspot (green circle) and the current (0 Ma) global distribution of active and inactive hotspots (blue)^69^. The size of the hotspot circle corresponds to the magnitude of the anomalous mass flux^69^. NA = North America Cordilleran; SA = South American Cordilleran; NE = New England; ICS = Indochina-Sumatra; JK = Jiangda-Hoh Xil Shan-Karakorum; MO = Mongol-Okhotsk; WP = West Pacific.

The paleogeographic location of Wrangellia is constrained by various paleomagnetic studies (Fig. 2a). Yole and Irving^43^ investigated the paleomagnetism of the Karmutsen Formation and concluded that the paleolatitude of Wrangellia is 18 ± 6° S or 18 ± 6° N. This would imply a paleolatitude of 12° S to 24° S or 12° N to 24° N. They also compared the results to those from Schwarz et al.^44^ that determined paleolatitudes of 13 ± 15° and 17 ± 12° (which would be from 2° S to 39° N). Stone^45^ based on paleomagnetic mean poles suggests that Wrangellia was likely very close to, and probably north of the equator. Panuska^46^ also summarized that Wrangellia occupied a northern hemisphere position (10°–20° north latitude) during the late Paleozoic and early Mesozoic. Symons^47^, using paleomagnetic data from 46 sites (674 specimens) of the Crystalline Gneiss Complex on the west coast of Vancouver Island, indicates that the Wrangellia terrane was located at an 18 ± 6° S paleolatitude (i.e. 24° S to 12° S). Hillhouse and Gromme^48^ measured 46 Triassic lava flows to yield a mean paleolatitude of 13° N or 13.9° S. They also summarized that extensive sampling of the Triassic rocks of Wrangellia in Alaska and British Columbia has consistently yielded paleolatitudes of 10°–17°. Hillhouse and Coe^49^ summarized all the paleomagnetic data from Alaska (Wrangellia) and stated that “all parts of Wrangellia apparently originated within 18° of the Triassic equator”. The prevailing paleogeographic reconstructions of the early Mesozoic (ca. 230 Ma), e.g. Golonka^50^, Scotese and Schettino^51^, and Cao et al.^52^, all place Wrangellia close to the equator.

**Figure 3:**
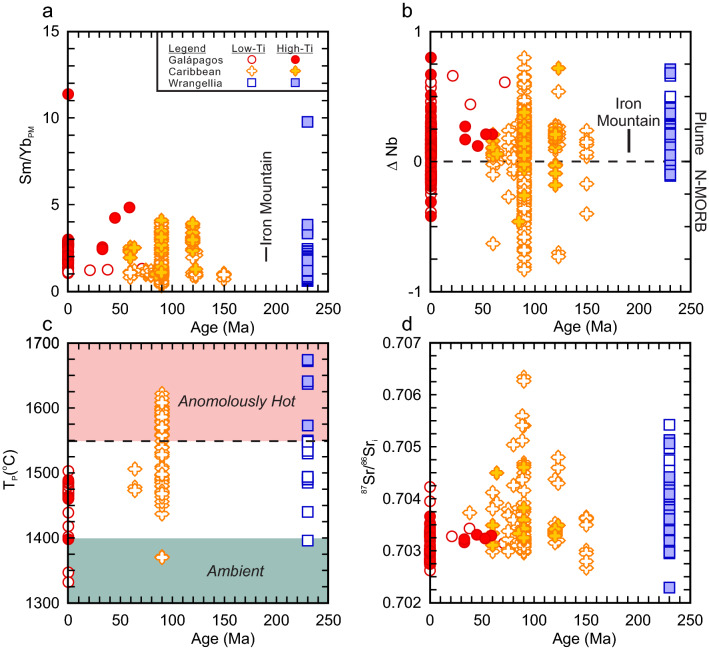

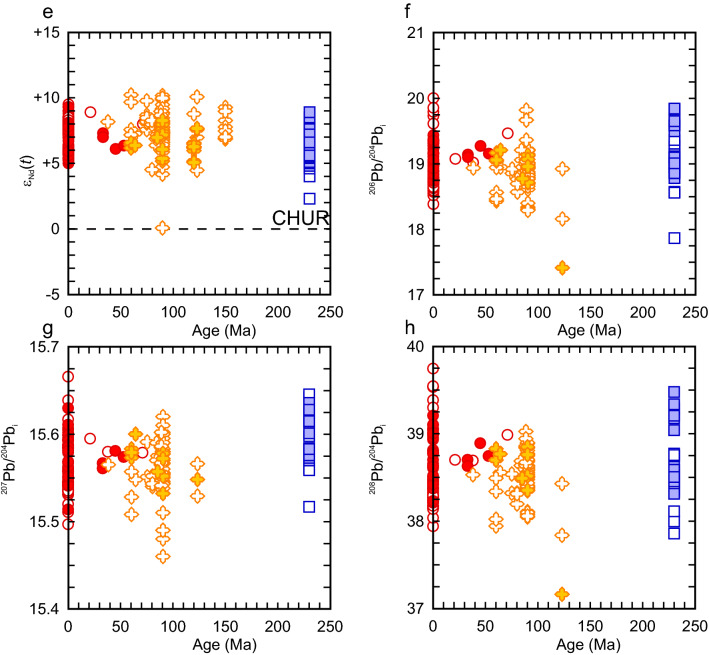
Secular geochemical comparison of the volcanic rocks of Wrangellia, Caribbean plateau, and Galápagos Islands using (**a**) Sm/Yb_PM_, (**b**) ΔNb, (**c**) calculated mantle potential temperature (T_P_ °C), (**d**) initial ^87^Sr/^86^Sr ratio, (**e**), ε_Nd_(*t*), (**f**), initial ^206^Pb/^204^Pb, (**g**) initial ^207^Pb/^204^Pb, and (**h**) initial ^208^Pb/^204^Pb. The range of the Early Jurassic Iron Mountain within-plate rocks from the Ingalls ophiolite are shown in panels a and b^54^. PM = normalized to primitive mantle values^55^. ΔNb = 1.74 + log[Nb/Y] − 1.92log[Zr/Y])^56^. If the age of the individual sample was not provided in the GEOROC database then it was assumed to be 230 Ma for the Wrangellia rocks, 90 Ma for the Caribbean rocks, and 0.01 Ma for the Galápagos Islands.

The flood basalts of Wrangellia (Nikolai and Karmutsen lavas) are variably altered but most are tholeiitic with a minor amount of mildly alkalic flows^13,15–18^. The Mg# of the basalts ranges from ~ 72 to ~ 27 indicating some rocks are compositionally primitive or near primitive (Mg# = 72–65) whereas others are differentiated (Mg# < 65). The basalts can be subdivided into high-Ti (≥ 1.4 wt%) and low-Ti (< 1.0 wt%) groups that reflect different sources^16^. Furthermore, high-Mg (MgO = 8 to 12 wt%; Mg# = 61–74) basalt and picrite (MgO = 13 to 20 wt%; Mg# = 70–78) are identified within the volcanic successions of the Karmutsen Formation. The high-Mg basalt and picrites are tholeiitic and have low-TiO_2_ (< 1.0 wt%) concentrations, and testify to a high temperature regime^18,53^. The primitive mantle normalized La/Yb_PM_ (0.4 to 12.9; avg = 2.1) and Sm/Yb_PM_ (0.6 to 9.8; avg = 1.5) ratios of all rock types are variable but generally low (Fig. 3a). Moreover, their ΔNb values (ΔNb = 1.74 + log[Nb/Y] − 1.92log[Zr/Y]), an indicator of source characteristics, are nearly all > 0 (ΔNb =  − 0.11 to 0.71; avg. = 0.16) and consistent with a mantle plume source^56^ (Fig. 3b). The Sr–Nd-Pb isotopes across all samples are similar but show some variability and indicative of a depleted to moderately depleted mantle source (^87^Sr/^86^Sr_i_ = 0.70229 to 0.70542; ε_Nd_(*t*) =  + 2.3 to + 9.0; ^206^Pb/^204^Pb = 17.868 to 20.297; ^207^Pb/^204^Pb = 15.517 to 15.646; ^208^Pb/^204^Pb = 37.858 to 39.478) (Fig. 3). It is thought that the low-Ti tholeiitic basalt is derived by small degrees (< 5%) of melting of the Paleozoic sub-arc lithospheric mantle that was high field strength element (HFSE)-depleted. In comparison, the high-Ti tholeiitic basalts, high-Mg basalts, and picrites are thought to be derived from a Pacific plume-type mantle source similar to the Caribbean Plateau^14,15,17,18^.

The accumulated fractional melting mantle potential temperature (T_P_) estimates of the basalt and picrite from Wrangellia range from 1674 to 1396 °C with the corresponding eruptive temperature estimates range from 1506 to 1300 °C (Fig. 3c). The high T_P_ estimates of some rocks are consistent with thermal conditions expected for a mantle plume^14,18,57^ and an oceanic plateau origin^14,15,17,18^.

### Spatial correlation of Wrangellia, the Caribbean plateau, and the Galápagos hotspot

The mafic and ultramafic rocks of Wrangellia are considered to be derived from a plume-type source that is similar to those which generated oceanic plateaux of the Pacific Ocean basin. The principal issue however, is the location of the hotspot that generated the volcanic rocks as the pre- and post-volcanic carbonate rocks of Wrangellia contain fossils that are typical of tropical-equatorial latitudes to mid-latitudes of the eastern Pacific (Panthalassa) Ocean^58–61^. Paleomagnetic data from different sections of Wrangellia consistently yield equatorial to near equatorial latitudes (± 18° of the equator) at the time of their eruption^47,48,50–52^. Furthermore, Belasky et al.^58^ suggests that Wrangellia was within 2000–3000 km from coastal North America during the Early Permian based on fossils and the location of the East Pacific (Panthalassa) barrier (EPB) but, it is likely that at the time of eruption during the Carnian-Norian that Wrangellia was even closer to coastal North America than during the Permian.

Torsvik et al.^62,63^ and Burke and Torsvik^64^ argue that the majority (~ 80%) of oceanic hotspots and continental flood basalt provinces emplaced since the Carboniferous are spatially correlated to long term stability of the 1% slow-velocity contour in the lowermost layer of the mean shear-wave tomographic model (SMEAN). The 1% contour defines a plume generation zone and is referred to as a large low shear velocity province (LLSVP). The current LLSVPs are primarily located beneath the African plate and the Pacific plate. It is at the boundary regions of an LLSVP that thermally anomalous upwelling of deep-seated mantle is thought to occur and manifests at the surface as oceanic islands/plateaux and continental flood basal provinces^62,63^. If this is the case, then the paleogeographic location of the Wrangellia flood basalts can be constrained by superimposing the current Pacific LLSVP on a Carnian plate reconstruction map of Pangea as the African LLSVP was too far to the east at the time. The intersection of the paleomagnetic-derived latitudinal range of Wrangellia with the Pacific LLSVP and the EPB is outlined on Fig. 2a. The distances obtained from the intersection point of the Pacific LLSVP range from ~ 10,000 km at the farthest point from western North America to ~ 4200 km at the closest point. The farthest intersection point overlaps with the modern location of Hawaii but the closest intersection point is still outside the estimated distance of Wrangellia proposed by Belasky et al.^58^. However, it is likely that the LLSVPs are not fixed and can wander^65–68^. Thus, the mostly likely location of the hotspot that generated the Wrangellia volcanic rocks is within the latitudinal variation but between the current easternmost point of the ~ 1% contour of the current Pacific LLSVP and the region advocated by Belasky et al.^58^ (Fig. 2a).

**Figure 4:**
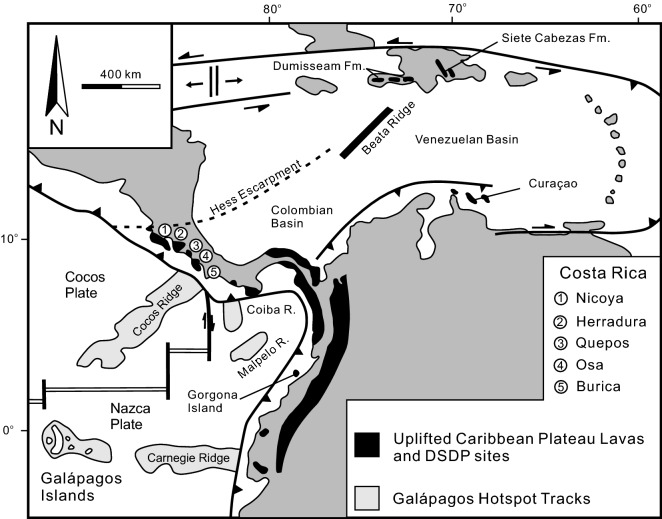
Distribution of the Caribbean plateau rocks and the locations of the Galápagos Islands, Carnegie Ridge, and Cocos Ridge^71^.

The only known and active hotspot that corresponds to the possible paleogeographic area of Wrangellia is the Galápagos hotspot (Fig. 2b)^69^. The Galápagos hotspot is located at the equator and just south of the active spreading centre separating the Cocos plate and the Nazca plate (Fig. 4). It is responsible for the present day Galápagos Islands and has been active for at least 20 million years as submerged volcanic edifices along the Cocos and Carnegie Ridges can be traced back to their point of origin^70^. The Galápagos hotspot is also linked to the generation of mafic (alkaline and tholeiitic) and ultramafic (komatiites) volcanic rocks of the Caribbean Plateau (Fig. 4) at ~ 90 Ma and ~ 70 Ma but may stretch back to 140–110 Ma^72–80^. Some kinematic plate reconstructions suggest the Caribbean Plateau developed 1000–3000 km east of the Galápagos hotspot whereas others indicate there is a spatial–temporal correlation^81–85^. The correlation between the paleogeographic eruption location of the Wrangellia flood basalt and the current Galápagos hotspot is intriguing and offers a possible explanation for the eruption of the Karmusten picrites and some primary basaltic lava as they require mantle potential temperatures > 1550 °C which is indicated for some Caribbean plateau rocks^18,53,57,78,86^. Moreover, a long-lived Galápagos hotspot model is supported by the Early Cretaceous (~ 140 Ma) basalt along the Nicoya Peninsula of Costa Rica and consistent with the development of the Caribbean Plateau by the accumulation of seamounts and oceanic plateaux at a subduction zone over a period of time rather than derivation by the initial plume head phase of the hotspot^75,78^.

The eruption of ultramafic (picrite and komatiite) volcanic rocks during the Phanerozoic is relatively rare (e.g., North Atlantic Igneous Province, the Caribbean plateau, and Emeishan large igneous province) and they are all considered to be attributed to a mantle plume or hotspot^57,78,87–91^. A comparison of the calculated mantle potential temperatures of Galápagos, Caribbean Plateau, and Wrangellia volcanic rocks using PRIMELT3 shows significant overlap but only the Caribbean Plateau and Wrangellia rocks extend to anonymously high estimates (Fig. 3c). Herzberg and Gazel^57^ and Trela et al.^92^ interpret the thermal decline from 90 to 70 Ma to the recent eruptions at the Galápagos Islands as evidence of a cooling trend in the hotspot related to elevated pyroxenite melt production.

Further support of the hotspot-association between the Wrangellia, Caribbean, and Galápagos volcanic rocks is their isotopic similarity. As previously noted by Greene et al.^16,18^, the total range of ^87^Sr/^86^Sr_i_, ε_Nd_(_t_), ^208^Pb/^204^Pb_i_, ^207^Pb/^204^Pb_i_, and ^206^Pb/^204^Pb_i_ values of Wrangellia volcanic rocks overlap with those of the Caribbean Plateau and the Galápagos Islands (Fig. 3d–h). Although the isotopic similarity cannot confirm ancestry from a specific mantle source or hotspot, it is still noteworthy that the isotopic compositions have a similar magnitude of internal variability as the rocks of the Hawaii-Emperor island chain^93^. Nevertheless, the elevated mantle potential temperatures, similar paleogeographic eruptive locations, and the rarity of Phanerozoic ultramafic lavas are compatible with a single, albeit isotopically heterogeneous, source hypothesis^16–18,53,57,72,90,92,94,95^.

### The eastern Pacific hotspot region and possible Mesozoic hotspot track

**Figure 5:**
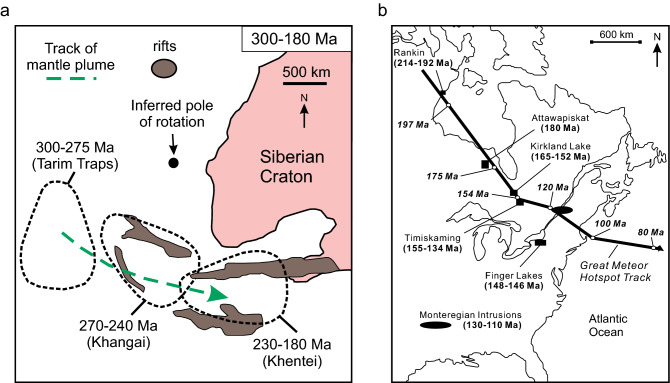
(**a**) The proposed Mongolia mantle plume track of Central Asia^101^. (**b**) The distribution of magmatic rocks correlated to the Great Meteor hotspot track and the position of North America^100^.

The longevity of magmatism at an oceanic hotspot is unknown but the Hawaiian hotspot has likely been active for 100–150 million years^96^. Furthermore, the Louisville and Arago (Rurutu) hotspots may have been active for ~ 120 million years as well^97,98^. Mantle plume tracks within continental crust suggested for the Mongolia plume of Central Asia (~ 120 m.y.) and the Great Meteor hotspot track (~ 200 m.y.) of North America both exceed 100 million years^99–101^ (Fig. 5). The timeframe between the eruption of the youngest volcanic rocks of Wrangellia (~ 225 Ma) to the oldest rock (~ 140 Ma) considered to be related to the Caribbean plateau is ~ 85 million years^12,75^ and within the known lifespans of active hotspots but is also within the range of the Great Meteor hotspot track if extended to include the modern Galápagos Islands, and the Cocos and Carnegie ridges.

Such a large time gap and the orientations of the rocks of Wrangellia and the Caribbean plateau is inconsistent with the ‘continuous’ creation of oceanic islands and seamounts that is typified by the Hawaiian hotspot but also observed through the magmatic spatial–temporal progression of the Great Meteor hotspot track. There are three possibilities that can explain the apparent lack of a magmatic track that would ‘connect’ the Wrangellia and the Caribbean plateau rocks: (1) there is no track and the Wrangellia and Caribbean rocks are ‘unconnected’ and derived from temporally distinct hotspots that developed within the same geographical region, (2) the ‘missing’ island track was subducted, or (3) there is a track but, is has yet to be identified. The different orientations of the Wrangellia (north–south) rocks and Caribbean (west–east) rocks could be related to ridge jump, hotspot drifting, or both^62,102–106^.

The hypothesis that the Wrangellia and Caribbean plateau rocks, and by association the Galápagos Islands, are unrelated to the same hotspot and that no track was created is reasonable and perhaps the most likely scenario. However, there are two implications for the ‘unconnected’ hypothesis that would be unusual for oceanic hotspots. Firstly, the volcanic rocks of Wrangellia erupted over a short period from ~ 230 to ~ 225 Ma^12^. There is nothing unusual with such a short eruptive duration per se as it similar to some continental large igneous provinces but, nearly all oceanic hotspots have island chains that indicate long-lived magmatism, plume migration, and plate motion^67,69,107–110^. Therefore, it is unlikely that the hotspot responsible for the Wrangellia magmatism was short-lived and did not have a track. Secondly, regardless of the duration of magmatism, the high mantle potential temperature estimates indicate that the eastern Pacific/Panthalassa Ocean has been a region of anomalously hot mantle upwelling periodically for ~ 230 million years as the region also witnessed the eruption of picritic and komatiitic lavas of the Caribbean plateau. Consequently, it would appear that the eastern Pacific/Panthalassa Ocean was unique in this regard.

The subduction of the hypothetical ‘Wrangellia hotspot’ island track is possible as is it known that seamounts and oceanic islands enter the subduction zones of the Costa Rica margin, Aleutian margin, and Izu-Bonin margin^111,112^. In this case, the ‘missing’ seamounts and islands related to Wrangellia would be emplaced on an oceanic plate that was destroyed during eastward subduction beneath North America. Although the complete subduction of the island track is a possibility, many seamounts and oceanic islands are accreted to continental margins and commonly identified in collisional belts^113,114^. Thus, the circumstances that led to the accretion of Wrangellia to North America and not the associated island chain requires an explanation. One such explanation could be that Wrangellia was built upon an older, relatively buoyant substrate (e.g., arc basement), whereas the island track was built directly upon oceanic crust^13^. Regardless, if the island chain subducted then the verification of its existence is problematic.

**Figure 6:**
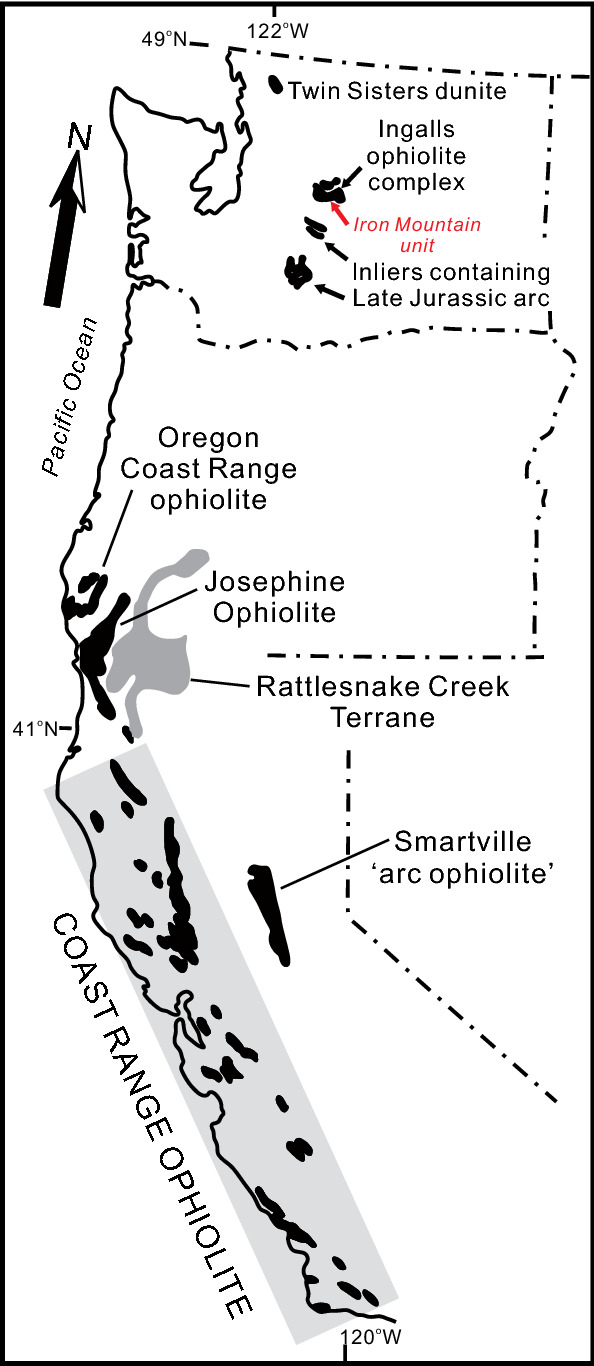
Distribution of Middle to Late Jurassic ophiolites of the North American Cordillera from Washington to California^118^.

**Figure 7:**
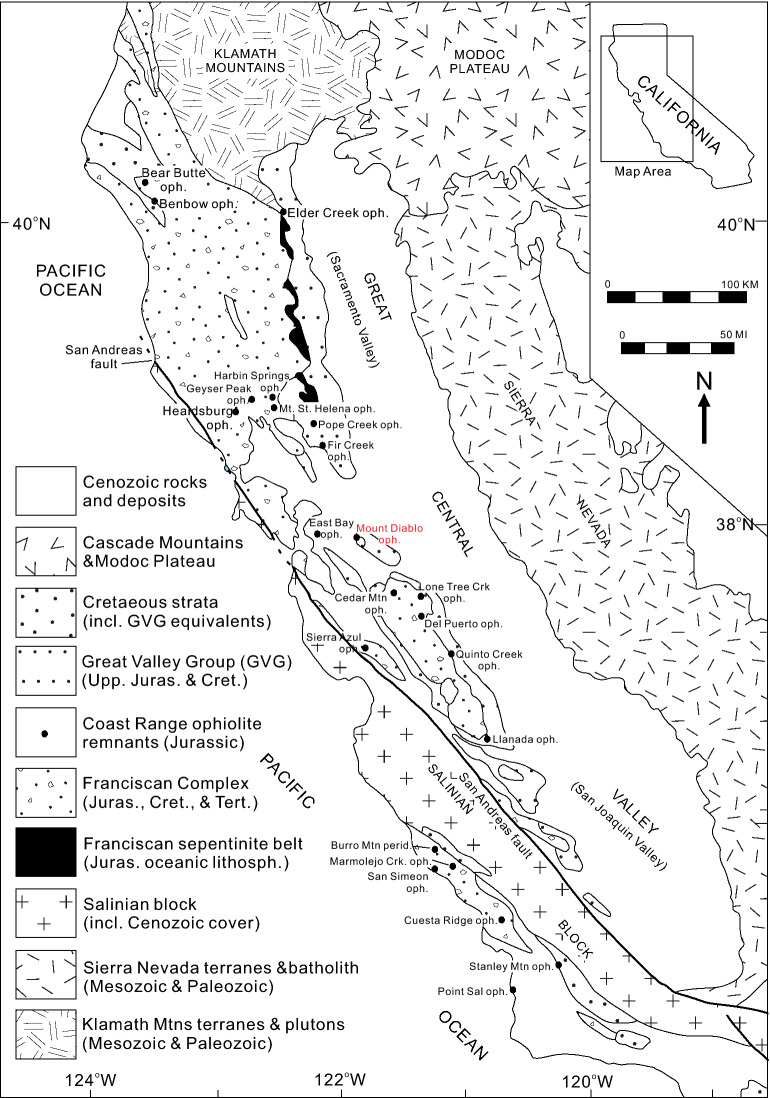
Simplified geological map of western California showing the locations and ages of the Middle to Late Jurassic and Coastal Range ophiolites^116^.

The third possibility is that there are uncorrelated units of the ‘Wrangellia hotspot’ track that accreted to western North America. From northern Washington to southern California there are a number of Jurassic ophiolitic units that have reported ages ranging from ~ 190 to ~ 160 Ma and include the Ingalls Ophiolite (Washington), Oregon Coast Range Ophiolite (Oregon), Josephine Ophiolite (Oregon-California), and the Coast Range Ophiolites of California^54,115,116^ (Fig. 6). The age of the northern ophiolites appears to decrease southward as the oldest reported age from the Ingalls Ophiolite (190–160 Ma) is Early Jurassic (192.1 ± 0.3 Ma) whereas the Josephine ophiolite (164–162 Ma) is Middle Jurassic^117–119^. The Coast Range Ophiolites of California do not show a definitive age progression but, east of the San Andreas Fault, the northern units (i.e., Elder Creek, Harbin Springs, Healdsburg, Mount Diablo; 172–163 Ma) tend to be older than the southern (Sierra Azul, Del Puerto, Llanada; 168–161 Ma) units^116^. To the west of the San Andreas Fault the ophiolite fragments (San Simeaon, Cuesta Ridge, Stanley Mountain, Point Sal; 166–160 Ma) are similar in age to the fragments south of Mount Diablo (Fig. 7).

The tectonomagmatic origins of the Jurassic ophiolites are a topic of considerable debate as there are three principal models proposed to explain their origin^54,115,116,120,121^. Ingersoll^120^ summarizes the tectonomagmatic models of the Coast Range Ophiolites and offers arguments in favour and against each one. The models are: (1) “formation by intra-arc and back-arc spreading related to an east-facing intraoceanic arc” that collided with a westward oriented continental margin arc during the Kimmeridgian to Tithonian; (2) “formation by open-ocean seafloor spreading” and their subsequent “incorporation into the continent margin during trench initiation outboard of an existing continental-margin trench”, and (3) “formation by forearc oblique rifting along the continental margin, followed by partial closure”. Although the models are conflicting, all consider the ophiolites to be principally derived from oceanic lithosphere that developed by melting and emplacement at spreading centres and not hotspot related^115^.

The compositions of the basaltic rocks of the ophiolites are mostly similar to mid-ocean ridge basalt and island-arc tholeiites but there are within-plate compositions reported from the Ingalls and Coast Range ophiolites^54^. Of particulate interest is the Early Jurassic (192.1 ± 0.3 Ma) Iron Mountain unit of the Ingalls ophiolite as it is interpreted to represent an off-axis seamount that erupted on older ocean crust before accretion to North America^118,119^ (Fig. 7). Radiogenic isotopes are not available from the Iron Mountain unit and the rocks are not suitable for PRIMELT3 calculations but, the ΔNb values (0.10 to 0.25) and other trace element ratios (e.g., Sm/Yb_PM_, Th/Nb_PM_, Zr/Nb, Dy/Dy*) are within the range of the Wrangellia, Caribbean plateau, and Galápagos Islands rocks. The fact that the Iron Mountain unit has within-plate compositions, is south of Wrangellia, and is younger than the Karmutsen Formation indicates that it could be supportive evidence of the relict hotspot track hypothesis.

## Conclusions

The Triassic volcanic rocks of Wrangellia are considered to be derived from a Pacific-type mantle plume source. The exact location of the hotspot is uncertain but fossil and paleomagnetic data indicate that it was located at equatorial to topical latitudes of the eastern Pacific (Panthalassa) ocean. The paleogeographic location of the Wrangellia hotspot is within the region of the current Galápagos hotspot. A comparison of the mantle potential temperature estimates, trace element geochemistry, and Sr–Nd–Pb isotopes between the volcanic rocks of Wrangellia, Caribbean plateau, and the Galápagos Islands shows significant overlap and the geochemical variability is similar to other oceanic island chains (e.g., Hawaii-Emperor island chain). Our model necessitates that the potential Wrangellia-Caribbean-Galápagos hotspot was active for ~ 230 million years which is within the range of activity for the Great Meteor hotspot. The apparent absence of a confirmed hot spot track argues against a direct connection between the Late Triassic Wrangellia volcanic rocks and the Early Cretaceous initial flows of the Caribbean plateau but, it is possible that Early to Middle Jurassic oceanic islands/seamounts were either subducted or accreted to North America (e.g., Iron Mountain unit of the Ingalls Ophiolite). The evidence of a link between the volcanic rocks of Wrangellia, Caribbean plateau, and Galápagos Islands to a common, long-lived hotspot is compelling and cannot be easily dismissed. The Wrangellia-Caribbean-Galápagos connection is possible and they are either related to a single, long-lived equatorial hotspot or, more broadly, that the equatorial region of the eastern Panthalassa/Pacific Ocean, near the Americas, has been a region of anomalously hot mantle upwelling for ~ 230 million years. If our model is correct, then the Galápagos hotspot is the longest continually active hotspot of the Phanerozoic. The primary issues that must be resolved are tighter paleomagnetic constraints on Wrangellia volcanic rocks and the discovery of more Early to Middle Jurassic rocks that are OIB-like within the North American Cordillera south of British Columbia.

## Methods

Geochemical data of the Wrangellia, Caribbean plateau, and Galápagos Islands volcanic rocks was compiled using GEOROC (http://georoc.mpch-mainz.gwdg.de/georoc/) and can be found as supplementary table S1. The primary melt compositions and mantle potential temperature estimates were calculated using PRIMELT3^120^. The major elemental data of each sample was entered into PRIMELT3 and calculated using an Fe_2_O_3_/TiO_2_ ratio of 0.5 and 1.0, pressure of 1 bar, H_2_O = 0 wt% and the lowest possible FeO content. The rock compositions and accumulated fractional melting (AFM) results are reported in table S2.

## Supplementary Information

Supplementary Tables.
